# Association between social support and depressive symptoms among Chinese nurses with formal employment versus contract-based employment

**DOI:** 10.3389/fpsyt.2023.1037499

**Published:** 2023-02-27

**Authors:** Chang Fu, Xuedan Cui, Lei Geng, Fenglin Cao

**Affiliations:** ^1^Department of Health Service and Management, School of Public Health and Management, Binzhou Medical University, Yantai, Shandong, China; ^2^Office of Physician Training, Yidu Central Hospital of Weifang, Weifang, Shandong, China; ^3^Department of Pediatric Surgery, Binzhou Medical University Hospital, Binzhou, Shandong, China; ^4^Department of Health Psychology, School of Nursing and Rehabilitation, Cheeloo College of Medicine, Shandong University, Jinan, Shandong, China

**Keywords:** social support, depressive symptoms, employment type, inequality, nurse

## Abstract

**Background:**

Inequalities may exist in social and health status among nurses with different employment types. Few studies have investigated the relationship between social support and depressive symptoms among formally employed nurses compared with those in contract-based employment. This study aimed to examine the associations between social support and depressive symptoms among Chinese nurses with different forms of employment.

**Methods:**

The present cross-sectional study was performed with 1,892 nurses from 12 tertiary hospitals in Shandong Province, China. The Social Support Rating Scale and the 10-item Center for Epidemiologic Studies Depression Scale were used to measure social support and depressive symptoms, respectively. The association between social support and depressive symptoms among participants was explored using multiple linear regression analysis.

**Results:**

The prevalence of depressive symptoms was 45.7%. The mean score for total social support was 40.16 (SD = 7.47), which was lower than the norms in the general Chinese population. Formally employed participants’ total social support scores were statistically significantly higher than those of contract-based employees (*p* ≤ 0.05). After controlling for confounding factors, the multiple linear regression analysis showed that subjective support and support utilization scores were inversely associated with depressive symptoms. Objective support scores were negatively associated with depressive symptoms only among contract-employment nurses.

**Conclusion:**

Chinese nurses have a higher prevalence of depressive symptoms and lower social support than the Chinese general population. Compared with contract-employment nurses, formally employed nurses had higher social support. Inverse associations exist between social support and depressive symptoms among nurses with different types of employment. It is suggested that improving Chinese nurses’ social support levels and reducing their depressive symptoms, especially for nurses employed through contracts, are critical.

## Introduction

Nurses who experience a high intensity of work and work-related pressure are generally prone to suffer from heavy work stress and burnout, which can lead to depression (1). Depression is a multidimensional disorder and has several negative effects on an individual’s health outcomes (2). Depression can harm an individual’s work performance, interpersonal and social communication, and quality of life (3). Depressive symptoms not only affect nurses’ health status but may also impact patients’ quality of care (2). Worldwide, there is a high occurrence of depressive symptoms among nurses. Previous studies found that 32.4% of Australian nurses, 18% of American nurses, and 43.83% of Chinese nurses experienced depressive symptoms (3–5). Therefore, hospital administrators and scholars worldwide should pay attention to nurses’ mental health.

In recent years, investigations on the relationship between social support and mental health have shown that social support has a protective effect on mental health. Social support is significantly associated with recovery from post-traumatic stress disorder (6), and older adults with good social support have a lower incidence of depression (7). Social support refers to the existence or availability of people one can rely on and from whom one can experience love, care, and value (8). In developed countries, medical professionals are well-respected and often have a high level of social support. The intensive relationship between medical professionals and patients needs to improve in China (9). Currently, the level of social support available to Chinese nurses is unknown because few surveys have investigated the relationship between social support and depressive symptoms for this group. Social support, as a multidimensional concept, consists of three dimensions: objective support, subjective support, and support utilization (6, 10). Different types of social support may have varied effects on individuals’ health (9), and it is unclear which types of social support are protective against depressive symptoms among nurses.

Although the nature of nursing employment varies by country, it is usually divided into permanent employment and fixed-term contract-based employment. For example, in Europe, permanent nurses are part of the primary labor market, and they work on an indefinite basis with good working conditions and development opportunities (11). By contrast, nurses employed through contracts are part of the secondary labor market and often experience poor working conditions, including job insecurity, low wages, and few benefits (11). Similarly, in China, nursing employment can be divided into “*bianzhi”* (permanent/formal employment) and contract-based jobs (12). The Chinese public often considers “*bianzhi”* jobs as formal employment guaranteed by the government, from which an individual employer cannot dismiss the incumbent. “*Bianzhi”* nurses have a steady income and certain benefits, including housing, health insurance, pension, etc. By contrast, nurses in contract-based positions are hired by the hospital; they do not have lifetime employment and might experience lower incomes and limited benefits (12). Previous studies have found that contract-employment nurses experience higher work stress and lower levels of organizational justice (13), which indicates that nurses with different employment types may have different levels of social support and health outcomes. However, few studies have compared the different associations between social support and depressive symptoms among formally employed (*bianzhi*) nurses and contract-employment nurses in China.

Therefore, this study aimed to: (1) examine the level of social support among Chinese nurses; (2) investigate the prevalence of depressive symptoms among Chinese nurses; and (3) investigate the associations between social support and depressive symptoms among formally employed nurses and contract-employment Chinese nurses.

## Materials and methods

### Study design and participants

From 30 July to 30 September 2020, a cross-sectional questionnaire survey was conducted among nurses in Shandong Province, China. Shandong Province, located in eastern China, which has 16 prefecture-level cities with a population of 100.7 million. It is a typical province in China in terms of population demographics, society, and culture (14). The survey adopted a multistage random sampling method. First, the prefecture-level cities were divided into high, medium, and low groups based on *per capita* GDP in 2019. Second, two prefecture-level cities were randomly chosen from each group and two tertiary hospitals were randomly selected from each of the two cities. Third, two-thirds of the departments were selected from internal medicine, surgery, obstetrics and gynecology, pediatrics, emergency, and others in each sampled hospital. Administrative and logistics departments were excluded. A questionnaire survey was administered to nurses in the selected departments. All information was collected based on nurses’ self-report. The inclusion criteria included voluntary participation, registration as a nurse, and being employed by the hospital. The exclusion criteria were as follows: nurses who were on vacation or who were participating in a continued education study course in another hospital, and persons with severe mental or physical impairments that would prevent them from participating (15). A total of 1,933 nurses participated in this study. After excluding those with missing data, 1,892 questionnaires were included in the analysis, showing an effective rate of 97.9%.

### Measurements

#### Depressive symptoms

A 10-item Center for Epidemiologic Studies Depression Scale (CESD-10) was used to measure depressive symptoms. The CESD-10 is a simplified version of the Center for Epidemiological Studies Depression Scale (CESD) revised by Andresen in 1994 (16). It has high reliability and validity within the Chinese population (17). The CESD-10 measures the extent to which an individual has experienced depressive symptoms in the past 7 days. Answers for each item include rarely (<1 days), some (1–2 days), occasionally (3–4 days), and most of the time (5–7 days). The total score ranges from 0 to 30, with higher scores suggesting higher levels of depressive symptoms. The cutoff score to identify individuals with depressive symptoms was 10 (16). In this study, Cronbach’s alphas for the CESD-10 were 0.696 and 0.674 in the formal employee and contract-based employee groups, respectively.

#### Social support

The Social Support Rating Scale (SSRS) was used to measure social support. The Chinese version of the SSRS, which was developed by Professor Xiao Shuiyuan, has been widely used in China (6, 18). The scale comprises 10 items and includes three dimensions: subjective support, objective support, and support utilization. Subjective support refers to the emotional support that an individual experiences and is closely related to their subjective feelings. An example of a question covering subjective support is: “How many friends do you have and how much support and help can they provide you?” Objective support refers to the actual support received by an individual. An example of a question covering objective support is: “What are your sources of financial support, and what helps you solve practical problems when you are in an emergency?” The utilization of support refers to an individual’s active utilization of various types of social support. An example of a question covering the utilization of support is: “How do you seek help when you have trouble?” The total score of the SSRS is the sum of the scores of subjective support, objective support, and support utilization, with higher scores indicating better levels of social support (9). In this survey, the Cronbach’s alpha for the SSRS was 0.79 in the formal employee group and 0.76 in the contract-based employee group.

#### Other variables

Demographic characteristics included age, sex, marital status, and educational background. Marital status was divided into married and single. Educational background was categorized as junior college or lower, bachelor’s degree, and master’s degree or higher. Professional characteristics included department, professional title, employment types, and working hours per week. The department was divided into internal medicine, surgery, obstetrics and gynecology, pediatrics, emergency, and others. Professional title was categorized as primary, intermediate, and senior. Employment type was divided into formal or contract-based employees. Working hours per week was divided into ≤40, 41–50, 51–60, and >60 h.

### Statistical analyses

All statistical analyses were conducted using SPSS 20.0. The distribution of depressive symptoms was analyzed using both the Kolmogorov–Smirnov test and histogram plot, which showed a normal distribution. The variance inflation factor (VIF) was used to test for multicollinearity in independent variables. The VIF was <10, indicating no multicollinearity (19). For descriptive statistics, continuous variables were described using means and standard deviations, and categorical variables were described using percentages. Variables were compared between formal employee and contract-based employee groups using independent *t* -tests for continuous variables and chi-square tests for categorical variables. Multiple linear regression analysis was used to examine the association between employment type and social support scores. Multivariate logistic regression analysis was used to examine the association between employment type and depressive symptoms. Multiple linear regression analysis was used to examine the association between social support and depressive symptoms among nurses. To describe the sensitivity analysis, the association between social support and depressive symptoms of participants was examined using multivariate logistic regression analysis. Statistical significance was set at <0.05.

### Ethical considerations

The present study was approved by the Ethical Review Committee of the School of Nursing and Rehabilitation, Shandong University (approval number: 2020-R-50). All participants provided informed consent for inclusion before participating in the survey.

## Results

### Sample characteristics

Table 1 shows the participants’ sociodemographic characteristics, work characteristics, status of depressive symptoms, and social support scores. The participants’ mean age was 33.9 years (*SD* = 7.3 years). Moreover, 93.9% were women, 80.1% were married, 88.1% had a bachelor’s degree, 39.3% worked in the surgical department, and nearly half had a primary professional title (47.4%) or an intermediate professional title (48.3%). More than half of the participants worked between 41 and 50 h per week (68.0%). The majority (78.3%) were contract-based employees. The mean CES-D 10 score was 9.17 (SD = 5.39), and 45.7% of the participants had depressive symptoms. The prevalence of depressive symptoms among contract-based employees was significantly higher than among formal employees (47.3% vs. 40.1%). The mean scores for total social support, subjective support, objective support, and support utilization were 40.16 (SD = 7.47), 24.04 (SD = 4.91), 8.28 (SD = 2.38), and 7.84 (SD = 1.98), respectively. Differences in age, sex, marital status, educational background, department, professional title, work hours per week, scores of social support, and depressive symptoms between the formal and contract-based employees were also statistically significant (*p* < 0.05). After controlling the possible confounding factors, the multiple regression analysis showed that there was a statistically significant association between the employment type and social support scores (formal employees have a higher level of social support than contract employees; Supplementary Table 1), while the association between employment type and depressive symptoms was not statistically significant (Supplementary Table 2).

**Table 1:** Demographic characteristics according to employment type.

Characteristics	Sample (*n* = 1892)	Formal employee (*n* = 411)	Contract employee (*n* = 1,481)	*t/χ* ^2^	Value of *p*
Age, years, mean ± SD	33.91 ± 7.27	42.78 ± 7.50	31.45 ± 4.91	28.975	<0.001
Sex, (%)				19.617	<0.001
Male	6.1	1.5	7.4		
Female	93.9	98.5	92.6		
Marital status, (%)				56.245	<0.001
Married	80.1	93.2	76.5		
Single	19.9	6.8	23.5		
Education background, (%)				196.141	<0.001
Junior college or less	7.2	3.2	8.3		
Bachelor	88.1	79.3	90.5		
Master or above	4.8	17.5	1.2		
Department, (%)				27.591	<0.001
Internal medicine	28.2	30.7	27.5		
Surgery	39.3	43.8	38.1		
Obstetrics and gynecology	5.0	4.6	5.1		
Pediatrics	7.1	7.3	7.1		
Emergency	8.7	2.4	10.4		
Other	11.6	11.2	11.7		
Professional title, (%)				522.137	<0.001
Primary	47.4	6.3	58.7		
Intermediate	48.3	74.7	41.0		
Senior	4.3	19.0	0.3		
Work hours per week, (%)				9.139	0.028
≤40	8.8	11.2	8.1		
41–50	68.0	69.8	67.5		
51–60	13.8	12.4	14.2		
>60	9.5	6.6	10.3		
Total social support scores, mean ± SD	40.16 ± 7.47	41.99 ± 7.61	39.65 ± 7.35	5.684	<0.001
Subjective support scores, mean ± SD	24.04 ± 4.91	24.91 ± 4.93	23.80 ± 4.89	4.069	<0.001
Objective support scores, mean ± SD	8.28 ± 2.38	9.01 ± 2.36	8.07 ± 2.35	7.149	<0.001
Support utilization score, mean ± SD	7.84 ± 1.98	8.07 ± 2.12	7.77 ± 1.93	2.572	0.010
Depressive symptoms, (%)				6.571	0.010
Yes	45.7	40.1	47.3		
No	54.3	59.9	52.7		
Depressive scores, mean ± SD	9.17 ± 5.39	8.27 ± 5.91	9.41 ± 5.22	−3.555	<0.001

SD, Standard deviation.

### The differences between social support scores among nurses and norms in the general Chinese population

Tables 2, 3 show the differences in social support between nurses in our sample and the norms in the general Chinese population. The formal employees’ scores for total social support, objective support, and support utilization were statistically significantly lower than the norms in the general population (20) (*p* < 0.05). Formal employees’ subjective support scores were statistically significantly higher than those of the general Chinese population (*p* < 0.05). Contract-based employees’ scores for total social support, objective support, and support utilization were statistically significantly lower than the norms in the Chinese general population (20) (*p* < 0.05).

**Table 2:** The difference of social support scores between formal employee and the norms of Chinese general population.

Variables	Formal employee	Norms of general population	*t*	Value of *p*
Total social support score, mean ± SD	41.99 ± 7.61	44.38 ± 8.38	−6.367	<0.001
Subjective support score, mean ± SD	24.91 ± 4.93	23.81 ± 4.75	4.523	<0.001
Objective support score, mean ± SD	9.01 ± 2.36	12.68 ± 3.47	−31.526	<0.001
Support utilization score, mean ± SD	8.07 ± 2.12	9.38 ± 3.40	−12.527	<0.001

**Table 3:** The difference of social support scores between contract employee and the norms of Chinese general population.

Variables	Contract employee	Norms of general population	*t*	Value of *p*
Total social support score, mean ± SD	39.65 ± 7.35	44.38 ± 8.38	−24.766	<0.001
Subjective support core, mean ± SD	23.80 ± 4.89	23.81 ± 4.75	0.079	>0.05
Objective support score, mean ± SD	8.07 ± 2.35	12.68 ± 3.47	−75.494	<0.001
Support utilization score, mean ± SD	7.77 ± 1.93	9.38 ± 3.40	−32.103	<0.001

### Multiple linear regression analysis

Table 4 reveals the associations between the three dimensions of social support and depressive symptoms among formal and contract-based participants. After adjusting for all covariates, the findings showed that among formal employees, subjective support and support utilization scores were inversely associated with depressive symptoms (subjective support: *β* = −0.237, SE = 0.064, *p* < 0.001; support utilization: *β* = −0.824, SE = 0.137, *p* < 0.001). Among contract-based employees, subjective support, objective support, and support utilization scores were also inversely associated with depressive symptoms (subjective support: *β* = −0.284, SE = 0.031, *p* < 0.001; objective support: *β* = −0.291, SE = 0.063, *p* = 0.035; support utilization: *β* = −0.559, SE = 0.070, *p* < 0.001). However, there was no statistically significant correlation between objective support scores and depressive symptoms among those with formal employment (*p* > 0.05). In the sensitivity analyses, the results of the multivariate logistic regression model showed the same associations between the three dimensions of social support and depressive symptoms among both formal and contract-based employees (Supplementary Table 3).

**Table 4:** Multiple linear regression model testing the association between social support and depressive symptoms among formal employee and contract employee.

Variables	Formal employee			Contract employee		
	*β* (SE)	95% CI of *β*	Value of *p*	*β* (SE)	95% CI of *β*	Value of *p*
Subjective supports score	−0.237(0.064)	−0.362 to −0.111	<0.001	−0.284 (0.031)	−0.345 to −0.223	<0.001
Objective supports score	−0.132(0.129)	−0.386 to 0.122	0.308	−0.291 (0.063)	−0.414 to −0.168	<0.001
Support utilization score	−0.824(0.137)	−1.093 to −0.554	<0.001	−0.559 (0.070)	−0.695 to −0.422	<0.001
Age	−0.062(0.044)	−0.150 to 0.025	0.163	0.076 (0.035)	0.008 to 0.143	0.029
Sex (ref. Male)						
Female	1.203(2.106)	−2.938 to 5.344	0.568	0.650 (0.481)	−0.293 to 1.593	0.176
Marital status (ref. Married)						
Single	−0.141(1.072)	−2.249 to 1.968	0.896	1.871 (0.380)	1.124 to 2.617	<0.001
Education level (ref. Junior college or less)						
Bachelor	1.167(1.464)	−1.711 to 4.044	0.426	−0.688 (0.444)	−1.559 to 0.183	0.122
Master or above	0.614(1.604)	−2.540 to 3.768	0.702	−1.574 (1.204)	−3.935 to −0.787	0.191
Department (ref. Other)						
Internal medicine	1.482(0.878)	−0.244 to 3.207	0.092	1.244 (0.436)	0.389 to 2.099	0.004
Surgery	1.691(0.837)	0.046 to 3.336	0.044	1.284 (0.415)	0.471 to 2.098	0.002
Obstetrics and gynecology	0.882(1.367)	−1.806 to 3.569	0.519	1.325 (0.648)	0.055 to 2.596	0.041
Pediatrics	3.549(1.193)	1.203 to 5.896	0.003	1.440 (0.589)	0.285 to 2.596	0.015
Emergency	1.539(1.779)	−1.959 to 5.037	0.388	0.898 (0.529)	−0.139 to 1.935	0.090
Professional title (ref. Primary)						
Intermediate	0.145(1.110)	−2.037 to 2.327	0.896	0.054 (0.295)	−0.525 to 0.632	0.855
Senior	−1.749(1.374)	−4.450 to 0.953	0.204	−3.888 (2.393)	−8.582 to 0.806	0.104
Weekly working hours (ref. ≤40)						
41–50	2.769(0.802)	1.192 to 4.347	0.001	−1.137 (0.449)	−2.017 to −0.256	0.011
51–60	2.972(1.038)	0.931 to 5.012	0.004	−0.155 (0.539)	−1.212 to 0.903	0.774
>60	6.004(1.259)	3.529 to 8.478	<0.001	−0.214 (0.576)	−1.344 to 0.917	0.711
∆*F*	10.885			23.903		
*R* ^2^	0.333			0.227		
Adjusted *R*^2^	0.303			0.218		

*β*, the coefficients; CI, confidence interval; SE, standard error.

## Discussion

This is the first study to investigate the association between different types of social support and depressive symptoms among Chinese nurses engaged in different forms of employment. As such, it provides useful information for health policymakers, hospital administrators, and nurses to consider when contemplating effective measures to prevent and reduce depressive symptoms.

In this study, the prevalence of depressive symptoms was 45.7%, which was higher than nurses in Guangdong province of China (37.59%) (3), and much higher than that in Chinese general population (12.6%) (21). Nurses have unique working conditions, as they often become overloaded with their clinical work; therefore, they experience long-term occupational pressure, which leads them to have poor mental health (3). Our results also showed that the prevalence of depressive symptoms among contract-based nurses was higher than that of formally employed nurses (47.3% vs. 40.1%). Therefore, the prevalence of depressive symptoms among nurses (especially contract-based nurses) requires the attention of hospital managers.

Our results showed that the level of social support among contract-based nurses was lower than that of formally employed nurses. The social inequalities between formally employed and contract-based nurses may explain this phenomenon (12). Individuals with higher social status may have more social resources and are easier to get social support. Our data also showed that the social support scores of both formally employed and contract-based nurses were significantly lower than those in the general Chinese population. There are three possible explanations for this phenomenon. First, in China, nurses often have a lower professional status than doctors; they are not valued by hospital administrators (22). Furthermore, nurses need to cope with tense nurse–patient relationships, and they seldom receive sufficient respect from patients (22). Second, nurses may face difficulty in balancing family roles with work (23); if nurses do not handle work–family conflicts, they may find it difficult to gain empathy from their work colleagues or other family members, leading to decrease social support. Third, because nurses work long hours, they may not have sufficient time to participate in social organizations; therefore, they have less social interaction (15). These explanations suggest that, to improve nurses’ social support, effective measures across society need improvement.

Subjective social support is a psychological perception of reality (24) that reflects an individual’s satisfaction with how they are supported, understood, and respected by others (9). Subjective support was a strong predictor of mental health improvement (25). Our results found that subjective support was negatively associated with depressive symptoms, indicating that nurses with more subjective support had fewer depressive symptoms than those who lacked subjective support. Individuals with more subjective support often have greater levels of satisfaction with their social support. A previous study reported that individuals who were more satisfied with their support were less likely to suffer from depression (26). In addition, subjective support can help individuals build a positive self-image and self-efficacy, which are protective factors for depressive symptoms (27). Thus, improving nurses’ subjective support can help to reduce their depressive symptoms.

A previous study demonstrated that nurses with higher objective support scores have genuinely received more support from their family members, government organizations, and social organizations (9). In this study, there was no significant association between the objective support scores and depressive symptoms of formally employed nurses. Objective assessment of received social support is less meaningful than subjective measures of social support, and it may have less effect on individuals’ mental health than subjective support (24). However, in this study, we found that objective support scores were inversely associated with depressive symptoms among contract-based nurses. There are two possible explanations for this observation. First, formally employed nurses have a higher occupational status in the hospital than contract-based nurses, as their position is guaranteed by the government, and they have extensive benefits (28). In addition, formally employed nurses often have higher educational levels, income levels, and social status (12). Therefore, formally employed nurses may take support from family members or social organizations for granted, whereas contract-based nurses may find themselves feeling grateful for this support owing to their relatively low status. A second possible reason is the treatment inequity between contract-based and formally employed nurses. A previous study has found that while both groups play an equal role in job responsibility, differences in treatment do exist (12). Such feelings of inequality can affect contract-based nurses’ work satisfaction, which may eventually lead to depression (29). Contract-based nurses who receive objective support may disregard their feelings of inequality, which can reduce the prevalence of depressive symptoms.

Our findings showed that social support utilization scores were inversely associated with depressive symptoms in both formally employed and contract-based nurses. Social support utilization reflects the degree to which individuals utilize available social support (6). According to the SSRS, higher support utilization scores indicate that a person may actively participate in social organizations (such as party, religious, or community organizations) and have many ways to seek help from others (such as family members, friends, or social organizations) when they experience trouble (9). Help from a varied use of social resources can help nurses overcome their troubles and relieve stress (30), which may, in turn, help them reduce their depressive symptoms (31, 32). Furthermore, participation in social organizations may promote social interactions among nurses and free them from stressful work, which would help them experience fewer depressive symptoms (33).

### Limitations

This study had some limitations. First, this was a cross-sectional study; therefore, causal relationships between social support and depressive symptoms among Chinese nurses could not be investigated. Second, the responses in this study were self-reported, which may have caused recall bias. Third, the participants in this study were all selected from tertiary hospitals; thus, it may not be possible to generalize the results to primary and secondary hospitals.

## Conclusion

Our study found that Chinese nurses have a higher prevalence of depressive symptoms than the Chinese general population. Formally employed nurses had a higher level of social support than contract-employment nurses. The level of social support for both formally employed and contract-employment nurses was lower than that in the general population. Both subjective support and support utilization scores were negatively associated with depressive symptoms among both formally employed and contract-based nurses. Objective support scores were negatively associated with depressive symptoms only for contract-based nurses. The findings of this study can be used to develop strategies to improve nurses’ social support and reduce depressive symptoms among them.

## Policy implications

To improve nurses’ mental health, our findings suggest that hospital administrators should pay attention to the role of nurses and arrange their working hours reasonably to reduce work pressure. They should also improve contract-based nurses’ benefits (e.g., housing, health insurance, and pensions). We also suggest that nurses’ family members should understand their unique working conditions, share housework, and take care of them. Policymakers should promote nurses’ contributions and foster a nurse-friendly social environment. Finally, we recommend that nurses should actively participate in social organizations to enhance their social interaction.

## Data availability statement

The data analyzed in this study is subject to the following licenses/restrictions: The datasets generated and/or analyzed during the current study are not publicly available due to agreements with participants who restricted data sharing but are available from the corresponding author on reasonable request. Requests to access these datasets should be directed to CF, fuchang@sdu.edu.cn.

## Ethics statement

The studies involving human participants were reviewed and approved by Ethical Review Committee of the School of Nursing and Rehabilitation, Shandong University. The patients/participants provided their written informed consent to participate in this study.

## Author contributions

CF contributed to the study design. CF, XC, and LG contributed to the data collection. CF contributed to the data analysis. CF, LG, and FC wrote the main manuscript text and revised the manuscript. All authors contributed to the article and approved the submitted version.

## Funding

This study was funded by the Surface Project of National Natural Science Foundation of China (grant number: 32071084). The funding source played no role in the design of this study; collection, analysis, and interpretation of data; writing of the report; or decision to submit the article for publication.

## Conflict of interest

The authors declare that the research was conducted in the absence of any commercial or financial relationships that could be construed as a potential conflict of interest.

## Publisher’s note

All claims expressed in this article are solely those of the authors and do not necessarily represent those of their affiliated organizations, or those of the publisher, the editors and the reviewers. Any product that may be evaluated in this article, or claim that may be made by its manufacturer, is not guaranteed or endorsed by the publisher.

## References

[1] Chen C Meier ST. Burnout and depression in nurses: a systematic review and meta-analysis. Int J Nurs Stud. (2021) 124:104099. doi: 10.1016/j.ijnurstu.2021.104099, PMID: 34715576

[2] Bai C Bai B Kong F. Strength use and nurses’ depressive symptoms: the mediating role of basic psychological needs satisfaction. J Nurs Manag. (2021) 29:1660–7. doi: 10.1111/jonm.13322, PMID: 33792987

[3] Xie N Qin Y Wang T Zeng Y Deng X Guan L. Prevalence of depressive symptoms among nurses in China: a systematic review and meta-analysis. PLoS One. (2020) 15:e0235448. doi: 10.1371/journal.pone.0235448, PMID: 32634150 PMC7340293

[4] Letvak S Ruhm CJ McCoy T. Depression in hospital-employed nurses. Clin Nurse Spec. (2012) 26:177–82. doi: 10.1097/NUR.0b013e3182503ef0, PMID: 22504476

[5] Maharaj S Lees T Lal S. Prevalence and risk factors of depression, anxiety, and stress in a cohort of Australian nurses. Int J Environ Res Public Health. (2018) 16:61. doi: 10.3390/ijerph16010061, PMID: 30591627 PMC6339147

[6] Dai W Chen L Tan H Wang J Lai Z Kaminga AC. Association between social support and recovery from post-traumatic stress disorder after flood: a 13-14 year follow-up study in Hunan. BMC Public Health. (2016) 16:194. doi: 10.1186/s12889-016-2871-x, PMID: 26924178 PMC4770534

[7] Tengku Mohd TAM Yunus RM Hairi F Hairi NN Choo WY. Social support and depression among community dwelling older adults in Asia: a systematic review. BMJ Open. (2019) 9:e026667. doi: 10.1136/bmjopen-2018-026667, PMID: 31320348 PMC6661578

[8] Liu L Gou Z Zuo J. Social support mediates loneliness and depression in elderly people. J Health Psychol. (2016) 21:750–8. doi: 10.1177/1359105314536941 24925547

[9] Fu C Wang G Shi X Cao F. Social support and depressive symptoms among physicians in tertiary hospitals in China: a cross-sectional study. BMC Psychiatry. (2021) 21:217. doi: 10.1186/s12888-021-03219-w, PMID: 33926402 PMC8082214

[10] Xiao S. The theoretical basis and research applications of the social support scale. J Clin Psychiatry. (1994) 4:98–100.

[11] Heponiemi T Kouvonen A Sinervo T Elovainio M. Do psychosocial factors moderate the association of fixed-term employment with work interference with family and sleeping problems in registered nurses: a cross-sectional questionnaire survey. Int J Nurs Stud. (2010) 47:1096–104. doi: 10.1016/j.ijnurstu.2010.01.008, PMID: 20188371

[12] Shang J You L Ma C Altares D Sloane DM Aiken LH. Nurse employment contracts in Chinese hospitals: impact of inequitable benefit structures on nurse and patient satisfaction. Hum Resour Health. (2014) 12:1. doi: 10.1186/1478-4491-12-1, PMID: 24418223 PMC3896777

[13] De Witte H Naswall K. Objective versus subjective job insecurity:consequences of temporary work for job satisfaction and organizational commitment in four European countries. Econ Ind Democr. (2003) 24:149–88. doi: 10.1177/0143831X03024002002

[14] Zhao S Zhang J Liu Y Ji H Lew B. The association between psychological strains and life satisfaction: evidence from medical staff in China. J Affect Disord. (2020) 260:105–10. doi: 10.1016/j.jad.2019.09.006, PMID: 31494361

[15] Fu C Ren Y Wang G Shi X Cao F. Fear of future workplace violence and its influencing factors among nurses in Shandong, China: a cross-sectional study. BMC Nurs. (2021) 20:123. doi: 10.1186/s12912-021-00644-w, PMID: 34233678 PMC8262060

[16] Andresen EM Malmgren JA Carter WB Patrick DL. Screening for depression in well older adults: evaluation of a short form of the CES-D (Center for Epidemiologic Studies Depression Scale). Am J Prev Med. (1994) 10:77–84. doi: 10.1016/S0749-3797(18)30622-6, PMID: 8037935

[17] Luo Y Zhu D Nicholas S He P. Depressive symptoms, health behaviors and risk of diabetes in Chinese mid-aged and older adults. J Affect Disord. (2019) 246:783–8. doi: 10.1016/j.jad.2018.12.131, PMID: 30623824

[18] Shi L Wang L Jia X Li Z Mu H Liu X. Prevalence and correlates of symptoms of post-traumatic stress disorder among Chinese healthcare workers exposed to physical violence: a cross-sectional study. BMJ Open. (2017) 7:e016810. doi: 10.1136/bmjopen-2017-016810, PMID: 28765135 PMC5642665

[19] Yang WC Lin CH Wang FC Lu MJ. Factors related to the improvement in quality of life for depressed inpatients treated with fluoxetine. BMC Psychiatry. (2017) 17:309. doi: 10.1186/s12888-017-1471-3, PMID: 28841824 PMC5574134

[20] Deng L Wang H Chen J Li L. Social support and negative emotion in parents of children with congenital heart disease before operation. Zhong Nan Da Xue Xue Bao Yi Xue Ban. (2013) 38:915–9. doi: 10.3969/j.issn.1672-7347.2013.09.008, PMID: 24071702

[21] Lancet. Mental health in China: what will be achieved by 2020? Lancet. (2015) 385:2548. doi: 10.1016/s0140-6736(15)61146-1 26122146

[22] Feng D Su S Yang Y Xia J Su Y. Job satisfaction mediates subjective social status and turnover intention among Chinese nurses. Nurs Health Sci. (2017) 19:388–92. doi: 10.1111/nhs.12357, PMID: 28636135

[23] Haji Matarsat HM Rahman HA Abdul-Mumin K. Work-family conflict, health status and job satisfaction among nurses. Br J Nurs. (2021) 30:54–8. doi: 10.12968/bjon.2021.30.1.54, PMID: 33433277

[24] Sun J Sun R Jiang Y Chen X Li Z Ma Z. The relationship between psychological health and social support: evidence from physicians in China. PLoS One. (2020) 15:e0228152. doi: 10.1371/journal.pone.0228152, PMID: 31995601 PMC6988930

[25] Lei X Kantor J. Social support and family functioning in Chinese families of children with autism Spectrum disorder. Int J Environ Res Public Health. (2021) 18:3504. doi: 10.3390/ijerph18073504, PMID: 33800586 PMC8037478

[26] Ren J Jiang X Yao J Li X Liu X Pang M. Depression, social support, and coping styles among pregnant women after the Lushan earthquake in Ya'an. PLoS One. (2015) 10:e0135809. doi: 10.1371/journal.pone.0135809, PMID: 26270035 PMC4535859

[27] Gyasi RM Phillips DR Abass K. Social support networks and psychological wellbeing in community-dwelling older Ghanaian cohorts. Int Psychogeriatr. (2019) 31:1047–57. doi: 10.1017/S1041610218001539, PMID: 30355385

[28] Bao M Huang C. Job preferences of medical and nursing students seeking employment in rural China: a discrete choice experiment. BMC Med Educ. (2021) 21:146. doi: 10.1186/s12909-021-02573-3, PMID: 33673842 PMC7934374

[29] Gao YQ Pan BC Sun W Wu H Wang JN Wang L. Depressive symptoms among Chinese nurses: prevalence and the associated factors. J Adv Nurs. (2012) 68:1166–75. doi: 10.1111/j.1365-2648.2011.05832.x 21950775

[30] Fu C Li Z Mao Z. Association between social activities and cognitive function among the elderly in China: a cross-sectional study. Int J Environ Res Public Health. (2018) 15:231. doi: 10.3390/ijerph15020231, PMID: 29385773 PMC5858300

[31] Hsieh HF Liu Y Hsu HT Ma SC Wang HH Ko CH. Relations between stress and depressive symptoms in psychiatric nurses: the mediating effects of sleep quality and occupational burnout. Int J Environ Res Public Health. (2021) 18:7327. doi: 10.3390/ijerph18147327, PMID: 34299778 PMC8303432

[32] Shepherd-Banigan M Bell JF Basu A Booth-LaForce C Harris JR. Workplace stress and working from home influence depressive symptoms among employed women with young children. Int J Behav Med. (2016) 23:102–11. doi: 10.1007/s12529-015-9482-2, PMID: 25894581

[33] Wang Y Li Z Fu C. Urban-rural differences in the association between social activities and depressive symptoms among older adults in China: a cross-sectional study. BMC Geriatr. (2021) 21:569. doi: 10.1186/s12877-021-02541-y, PMID: 34663230 PMC8522037

