# Supplementary material for: Association between social support and depressive symptoms among Chinese nurses with formal employment versus contract-based employment
Source: Front Psychiatry. 2023 Feb 27;14:1037499. doi: 10.3389/fpsyt.2023.1037499 (PMC10009186; doi:10.3389/fpsyt.2023.1037499)
Supplement: Supplementary file 1 [file Table_1.DOCX]

Supplementary Material

STable 1 Multiple linear regression model testing the association between employment type and social support among nurses

| Variable | B | SE | *P* |
| --- | --- | --- | --- |
| Employment type | 1.932 | 0.551 | **<0.001** |

Note:B, Coefficient; SE, Standard error. Model : adjusted for age, education background, department, professional title.

STable 2 Multivariate logistic regression model testing the association between social support and depressive symptoms among nurses

| Employment type | B | SE | *P* | OR | 95%CI |
| --- | --- | --- | --- | --- | --- |
| Formal (ref.) |  |  |  |  |  |
| Contact | 0.033 | 0.156 | 0.832 | 1.034 | 0.761~1.404 |

Note:B, Coefficient; SE, Standard error; OR, odds ratio; CI, confidence interval. Model : adjusted for age, education background, department, professional title.

STable 3 Multivariate logistic regression model testing the association between social support and depressive symptoms among formal employee and contract employee

| Type | Variables | B | SE | *P* | OR | 95%CI |
| --- | --- | --- | --- | --- | --- | --- |
| Formal | Subjective support | -0.094 | 0.031 | **0.003** | 0.910 | 0.856~0.968 |
|  | Objective support | -0.058 | 0.066 | 0.379 | 0.943 | 0.828~1.074 |
|  | Support utilization | -0.361 | 0.073 | **<0.001** | 0.697 | 0.605~0.804 |
|  |  |  |  |  |  |  |
| Contact | Subjective support | -0.112 | 0.015 | **<0.001** | 0.894 | 0.868~0.921 |
|  | Objective support | -0.120 | 0.030 | **<0.001** | 0.887 | 0.836~0.941 |
|  | Support utilization | -0.166 | 0.033 | **<0.001** | 0.847 | 0.793~0.905 |

Note:B, Coefficient; SE, Standard error; OR, odds ratio; CI, confidence interval. Model : adjusted for age, gender, marital status, education background, department, professional title, and work hours per week.
